# Supplementary material for: Age-Dependent Decline in Neuron Growth Potential and Mitochondria Functions in Cortical Neurons
Source: Cells. 2021 Jun 29;10(7):1625. doi: 10.3390/cells10071625 (PMC8306398; doi:10.3390/cells10071625)
Supplement: Supplementary file 1 [file cells-10-01625-s001.zip › cells-1241583-supplementary.pdf]

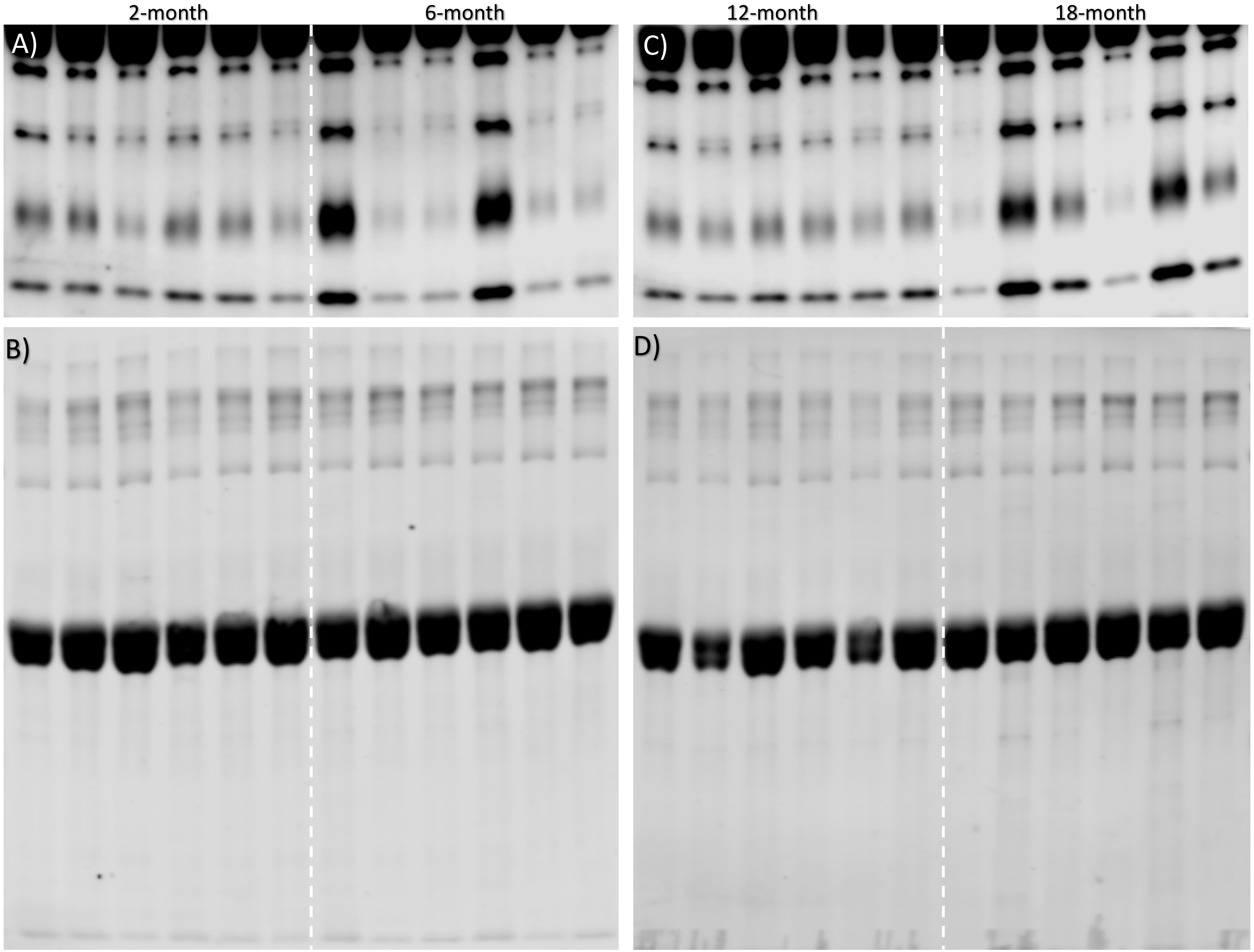

Supplementary Figure 1. No significant change in the Mitochondrial OXPHOS Complex protein expression in cortical neurons. Representative Western Blots of A) young (2- and 6-month) and C) older (12- and 18-month) cortical neurons (N= 3 samples/age) in duplicate, with their corresponding total protein stains, (B) and (D) respectively.
